# Human Organ-Specific 3D Cancer Models Produced by the Stromal Self-Assembly Method of Tissue Engineering for the Study of Solid Tumors

**DOI:** 10.1155/2020/6051210

**Published:** 2020-04-13

**Authors:** Vincent Roy, Brice Magne, Maude Vaillancourt-Audet, Mathieu Blais, Stéphane Chabaud, Emil Grammond, Léo Piquet, Julie Fradette, Isabelle Laverdière, Véronique J. Moulin, Solange Landreville, Lucie Germain, François A. Auger, François Gros-Louis, Stéphane Bolduc

**Affiliations:** ^1^Centre de Recherche du CHU de Québec-Université Laval, Axe Médecine Régénératrice, Québec, QC, Canada; ^2^Centre de Recherche en Organogénèse Expérimentale de l'Université Laval/LOEX, Québec, QC, Canada; ^3^Centre de Recherche sur le Cancer de l'Université Laval, Québec, QC, Canada; ^4^Department of Surgery, Faculty of Medicine, Université Laval, Québec, QC, Canada; ^5^Faculty of Pharmacy, Université Laval and CHU de Québec-Université Laval Research Center, Oncology Division, Québec, QC, Canada; ^6^Department of Ophthalmology, Faculty of Medicine, Université Laval, Québec, QC, Canada

## Abstract

Cancer research has considerably progressed with the improvement of *in vitro* study models, helping to understand the key role of the tumor microenvironment in cancer development and progression. Over the last few years, complex 3D human cell culture systems have gained much popularity over *in vivo* models, as they accurately mimic the tumor microenvironment and allow high-throughput drug screening. Of particular interest, *in vitro*human 3D tissue constructs, produced by the self-assembly method of tissue engineering, have been successfully used to model the tumor microenvironment and now represent a very promising approach to further develop diverse cancer models. In this review, we describe the importance of the tumor microenvironment and present the existing *in vitro* cancer models generated through the self-assembly method of tissue engineering. Lastly, we highlight the relevance of this approach to mimic various and complex tumors, including basal cell carcinoma, cutaneous neurofibroma, skin melanoma, bladder cancer, and uveal melanoma.

## 1. Introduction

Over the past years, various approaches have been employed to study cancer initiation, growth, and migration. Animal models have been instrumental in providing insight into the molecular mechanisms of tumor growth/proliferation and metastatic processes. However, studying cancer *in vivo* remains a considerable challenge nowadays, due to practical and ethical concerns, as well as to limitations in reliable predictions of human clinical trial outcomes [1]. Although current *in vitro* two-dimensional (2D) monolayer cell culture and conventional tridimensional (3D) cell culture systems have also led to significant advances in our understanding of tumor biology and the role of tumor microenvironment (TME), there are still several unmet needs to better model cancer invasion. The local TME is known to play a significant role in cancer progression and metastasis, where tumor cells can respond and adapt to a plethora of biochemical/biophysical signals from surrounding stromal cells and extracellular matrix (ECM) components [2]. In this regard, advances in tumor cell biology, 3D cell culture, and tissue engineering have enabled the rapid development of comprehensive *in vitro* tumor models with increased complexity, through the incorporation of multiple cell types. Furthermore, state-of-the-art tissue engineering technologies that incorporate endogenous patient-derived ECM proteins have emerged as unique alternatives to 3D bulk hydrogel and animal models to gain insights into the biological aspects of cancer development, which has not been fully possible using conventional culture systems. Other innovations including the incorporation of capillaries and ready-to-seed spheroids, grown under hypoxic or normoxic conditions, have led to precision medicine in the development of advanced tissue-engineered *in vitro* tumor models for patient-specific therapies, clinical management, and assessment of metastatic potential.

Among several protocols allowing the production of advanced exogenous material-free and patient-derived cancer models, the self-assembly method of tissue engineering distinguishes itself by the self-production and assembly of cell-specific endogenous ECM components. Indeed, it has been reported that ascorbate (vitamin C) can help mesenchymal cells produce their own ECM, to form a tissue highly similar to what is found *in vivo* [3]. Despite significant progresses over the last few years, there are still numerous challenges to create better models for various forms of primary and metastatic cancers, incorporating multicellular cultures and diverse cellular microenvironments capable of modulating ECM composition, cellular crosstalk, and distribution of soluble factors. In this review, we provide an overview of 3D cell culture models currently being employed with a particular focus on the stromal self-assembly method of tissue engineering, allowing the production of patient-derived organ- and human-specific models, for the study of diverse cancers.

## 2. Stroma and Tumor Microenvironment

### 2.1. Stromal Components

The stroma has long been viewed as a relatively inert structural support for organs. It is usually composed of connective tissue, the most abundant type of tissue in the body. It connects and supports other tissues and also plays a crucial role in organ development, homeostasis maintenance, and pathologies [4]. The connective tissue consists of cells, mainly fibroblasts, and ECM proteins. The specific ECM protein composition determines, in turn, the biochemical and biophysical properties influencing cell growth, differentiation, migration, and apoptosis [5–7].

The knowledge of the precise ECM composition of organ-specific human connective tissues is critical to better understand their structure-function relationship in healthy and diseased conditions. As evidenced for the skin dermis, the stroma can at least be divided into two distinct compartments: (1) the upper papillary dermis, a thin loosely arranged connective tissue, and (2) a deeper reticular dermis, consisting of a thick and dense irregular matrix [8]. These two distinct compartments have different biological roles but are also populated by distinct subsets of fibroblasts [9]. To accurately investigate pathological mechanisms, such as those occurring during cancer initiation and progression, generating innovative models including human organ-specific stroma has become essential. Among the cells inhabiting this rich environment, fibroblast is among the most abundant cell types present in the stroma, and is mostly responsible for the secretion and organization of the ECM.

Under stress conditions, fibroblasts adapt to their environment and have the ability to send local signals, to synthesize and reorganize the ECM of the skin and other organs [10, 11]. Fibroblast plasticity may be different depending on body parts, through a poorly understood mechanism involving the resident ECM framework and the microenvironment [12]. In certain pathological contexts, fibroblasts are capable of altering their cellular profile to become myofibroblasts, therefore producing massive amounts of ECM and contributing to organ dysfunction [13].

Many other cell types also live or transit through the stroma, including vascular endothelial cells, pericytes, adipocytes, and bone marrow stromal cells. Specialized cell types include nervous and immune cells. In addition to their classical roles, these cells can be unsuspected players in immune control [14] and wound healing [15]. Resident macrophages are especially sensitive to the matrix environment [16]. These cells could modify, directly or indirectly, through epithelial or endothelial cells, the secretion and organization of the ECM [17]. Some aspects of T cell memory function are now also associated with nonhematopoietic cells from the stroma [18].

### 2.2. Tumor Microenvironment

A plethora of different cell types located in the TME has been the focus of cancer research, including fibroblasts, myofibroblasts, endothelial cells, pericytes, macrophages, dendritic cells, and other immune cells. The nomenclature used to describe fibroblastic populations and fibroblast-like cells remains confusing because it widely varies in the literature. Most frequent terms include cancer-associated fibroblasts (CAFs), carcinoma-associated fibroblasts (also abbreviated as CAFs), tumor-associated fibroblasts, and tumor/cancer-associated stromal cells. A growing piece of evidence now shows that these cells are recruited from the stroma by cancer cells to promote ECM remodeling, neoangiogenesis, proliferation, invasion, migration, and metastasis, and mediate drug resistance mechanisms through the secretion of various growth factors, chemokines, and cytokines [19, 20]. Among these reactive stromal cells, CAFs are the most described and mainly derived from healthy fibroblasts, although they could also originate from mesenchymal stem/stromal cells (MSCs), adipocytes, endothelial cells, epithelial cells, or stellate cells [21]. They also share many similarities with wound healing-related myofibroblasts and differentiate when exposed to molecules such as transforming growth factor-beta, fibroblast growth factor 2, and platelet-derived growth factor [22]. CAFs are in constant interaction with cancer cells and have been shown to play a dual role in cancer progression, either promoting or suppressing it [23]. During tumor progression, CAFs enhance tumor growth through a variety of mechanisms, including ECM remodeling, and promote sustained inflammation via the increase of inflammatory cytokines, neoangiogenesis, and immune cell recruitment [24]. CAFs are now considered in many studies primary targets to limit cancer cell spreading [25].

Many cancers emerge from nonstromal tissues and migrate to the stroma, crossing the basal lamina in the case of epithelial cancers, or through intra- and extravasation processes in the case of circulating tumor cells [26]. The basal lamina is also present to delineate the endothelium, which separates two inner compartments, blood and connective tissues. It is composed of laminins [27], collagens (especially types IV and VII), and other molecules such as agrin, perlecan, and nidogens [28]. It is not only the physical support for epithelia and endothelia but also an important source of molecular cues that regulate cell interactions. Laminins are especially important because they anchor the cells to the basal lamina, through the cytoskeleton of epithelial cells or independently. They play a significant role as an interface between the epithelium and the stroma [28]. The presence of basal lamina is essential for the proper differentiation of epithelial cells, as evidenced by tissue engineering experiments where the inability of cells to deposit and assemble an organized basal lamina led to epithelial differentiation failures [29].

Other major constituents of the TME are ECM proteins. In healthy tissues, ECM is a dynamic microenvironment submitted to remodeling and degradation [30]. Deposited proteins include collagens, fibronectin [31], proteoglycans [32, 33], elastic fibres, and many others. They are mainly degraded by various enzymes including the matrix metalloproteinases (MMPs) [30]. In cancers, ECM is significantly modified to create an environment with different biophysical and mechanobiological features [34–36]. Cancer stroma is stiffer [37, 38], similar to what could be found in wound and fibrosis [39].It is also more prone to cell migration, due to a reduction of cell adhesion and epithelial-mesenchymal transition (EMT) [40].

### 2.3. Oxygen Tension in the Tumor Microenvironment

Each tissue is characterized by a specific oxygen partial pressure (pO_2_) in physiological conditions, namely, the *in situ* normoxia. This value varies from one tissue to another, as well as within the tissue itself, depending on the vasculature and the metabolic activity [41]. Tissue hypoxia is defined as an inadequate supply of oxygen that compromises biological functions. Since the uncontrolled proliferation of cancer cells causes tumors to outgrow their blood supply, most solid tumors have lower median pO_2_ than their tissue of origin [42]. Tumors are considered hypoxic when the pO_2_ falls below a critical value (8-10 mmHg) leading to a progressive decrease of the oxygen consumption rate or the ATP production rate in the tissue [43]. Adaptive responses to reduced oxygen availability are mediated by the hypoxia-inducible factors (HIFs), which are transcription factors active and stable under hypoxic conditions [44, 45]. HIFs function as master switches to induce the expression of several target genes involved in angiogenesis, cell survival, energy metabolism, differentiation, and invasion.

Although oxygen is supplied to the tissues and cells at reported concentrations of around 15-70 mmHg (2-9% O_2_) [46, 47], traditional tissue engineering practices rather use a pO_2_ that corresponds to the atmospheric level (160 mmHg or 21% O_2_). However, a physiologically relevant “low” pO_2_ could be beneficial for applications in tissue engineering [41]. For example, ECM synthesis and deposition by human dermal fibroblasts was shown to be optimal at a pO_2_ of 15 mmHg compared to cells exposed to a pO_2_ of 4 or 160 mmHg [48]. A low pO_2_ (15-40 mmHg) allowed the production of human vascularized cell sheets using the stromal vascular fraction of adipose tissue or MSCs without adding extrinsic growth factors [49, 50], as well as human tissue-engineered cartilaginous tissues using synovial MSCs without the use of an exogenous scaffold [51]. Also, stiffer intervertebral discs with increased glycosaminoglycan and collagen contents were generated when human MSCs were grown under hypoxic conditions (40 mmHg) during their 2D expansion and subsequent 3D culture [52].

Microenvironmental conditions such as oxygen tension and tissue dimensionality are critical determinants of tumor angiogenesis, fibrosis, and heterogeneity. Interestingly, many angiogenic factors and proteases that degrade or remodel the ECM, such as MMPs and lysyl oxidase, are among the genes comprising hypoxia-responsive elements in their promoter [53, 54]. Pathologically relevant 3D tumor invasion models, such as tumor spheroids with a hypoxic core embedded in self-assembled stromas, are thus required to better mimic the complex biophysical features of the TME and the tumor heterogeneity.

## 3. 3D Cell Culture and Spheroids as Cancer Models

2D cell culture has brought relevant information on cancer cell behavior and is still widely used for the prediction of drug responses, despite obvious limitations. Indeed, this simple and low-cost *in vitro* culture system has failed to properly model essential characteristics of tumors and TME such as the complex process of tumor invasion [55, 56]. 3D cell culture is an *in vitro* system that allows the growth and interaction of different cell types in a 3D scaffolding using either native ECM or biomaterials. Compared to conventional 2D cell cultures grown on plastic substrates, 3D systems better mimic the *in vivo* conditions in terms of cell-cell and cell-matrix interactions, physicochemical properties, and mechanical stresses [57–59]. 2D systems only offer weak interactions between cells and their microenvironment due to the inert nature of plastic. Moreover, cells grown as monolayers stay in a planar state, adhere and proliferate horizontally, and keep an apical-basal polarity [60, 61], which impedes or alters several biological processes, including differentiation and stemness. For example, few studies have demonstrated that freshly isolated cells can generate dedifferentiated cell populations when grown in 2D systems, while they can recover their native phenotype and primary functions when cultured in 3D environments [62, 63].

Interestingly, 3D culture systems can elicit a more physiological orientation of extracellular receptors, such as integrins and collagen receptors, for better interactions with neighbouring cells and ECM, compared to 2D systems [64]. They can also promote essential biological processes such as cell differentiation, migration, adhesion, proliferation, and morphogenesis [65]. While cells cultured in monolayers proliferate until they reach contact inhibition, cells in 3D develop in every direction and thus reach higher proliferation rates until homeostasis , especially when cocultured with stromal fibroblasts [66–68]. Consequently, 3D culture systems have progressively emerged as promising tools for the study of complex cellular interactions and now represent unavoidable models for clinical translation.

Due to the strong interaction between cancer cells and TME, 3D systems probably represent the best *in vitro* models for the study of cancers and the screening of innovative anticancer drugs. Therefore, 3D cultures may also offer a relevant platform to recreate and study the migratory behavior and invasion profile of cancer cells [69, 70], notably due to the expression of integrins and MMPs that resemble the native tissue [71]. Gene expression, mRNA splicing, and the biochemistry of cancer cells grown in 3D cultures are also similar to *in vivo* conditions [72, 73]. For example, the 3D culture of melanoma cells highly modulates gene expression with the notable upregulation of few *CXCL* genes, which have been linked to the progression and metastatic process of this cancer [72]. Moreover, 3D environments give rise to cancer cell behaviors that closely mimic those found *in vivo* [74–76]. Eventually, 3D cancer cell systems represent realistic models to monitor the effects of drug dosing and distribution [77].

Over the years, numerous 3D models have been developed to recreate the TME and study the cellular responses to drug treatments, including microfluidics, transwell-based assays, organoids, biogels, organs-on-a-chip, perfused cultures, bioreactors, and 3D bioprinting [78, 79]. These 3D models allow various degrees of complexity and may provide precious information on tumor formation, progression, and invasion, as reviewed elsewhere [80, 81]. More recently, 3D tumor spheroids have been extensively used in cancer research, especially in breast, lung, prostate, and colorectal cancer studies [81, 82]. These adaptative multicellular complex systems [83], composed of proliferative and quiescent cells, can reproduce highly interactive environments and closely mimic native tumor behaviors with similar histopathological features [82]. Depending on their size, they can carry a hypoxic or necrotic core, which is usually found in tumors [84]. Spheroidal structures also generate local nutrient, metabolite, and oxygen gradients, which help quantify the penetration of drugs [85].

3D tumor spheroids can be produced using various methods including the hanging drop technique and the bioreactor rotative system. The hanging drop technique can be used manually with an inverted dish or with specialized plates called ultralow attachment plates [82]. This method generates spheroids with rather similar sizes regardless of the cell line. However, this technique is time consuming, in spite of recent works speeding up the fabrication process [86]. In contrast, rotative systems allow the production of massive amounts of spheroids but are expensive and create heterogeneous populations of spheroids [82]. More complex techniques are available but require expensive equipment and trained staff. Among them, the production of spheroids within 3D-printed scaffolds has gained popularity, since it provides a complex 3D support. However, this approach sometimes integrates exogenous materials and causes shear stress on cells [87, 88]. In summary, the 3D tumor spheroid model is suitable for certain study purposes but can be limited by its production method, the capacity of aggregation, and maximal viable time *in vitro*. In the following sections, we will, therefore, focus on the alternative use of 3D tumor spheroids integrated into tissue-engineered cancer models.

## 4. The Self-Assembly Approach of Tissue Engineering

**Figure 1:**
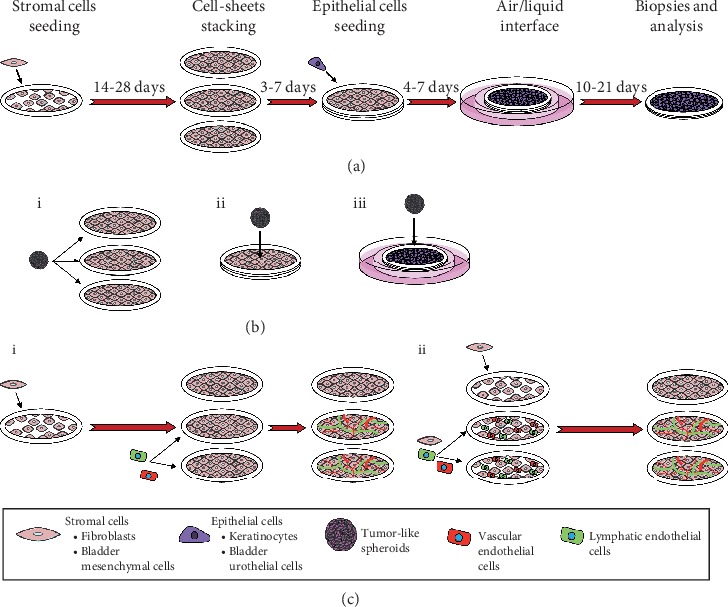
General description of the stromal self-assembly method for 3D cancer study. (a) Production steps and timeline of self-assembled constructs. Stromal cells are seeded and cultured during 14 to 28 days in the presence of ascorbate before the superimposition of the generated cell sheets to form a thicker stroma. Epithelial cells can also be seeded on the superimposed cell sheets before upraising the tissue-engineered construct at the air-liquid interface to induce epithelial differentiation. (b) Spheroid seeding time points. Spheroids can be seeded on single-cell sheets, before stacking (i), directly onto stacked stroma (ii), or on the well-formed epithelium (iii). (c) Microvascularization of stromal self-assembled constructs. Vascular endothelial cells (VEC) and/or lymphatic endothelial cells (LEC) can be seeded on the 2nd and 3rd matured stromal cell sheets few days before stacking (i) or coseeded with stromal cells on day 1 of the experimental protocol to form microvascularized single-cell sheets (ii). These self-assembled constructs can be analyzed using a plethora of techniques including histology, immunofluorescence, and transmission electron microscopy. Cells can be isolated for flow cytometry analysis, and total protein extracts can be collected for western blot and ELISA.

When cultured under optimal *in vitro* conditions, isolated primary stromal cells can constitutively secrete their own ECM proteins to promote the establishment of the cellular microenvironment. *In vivo*, stromal cells produce most of the ECM molecules that provide structural and biochemical support for surrounding cells [89]. We have taken advantage of the capacity of primary stromal cells to promote their microenvironment *in vitro* using the self-assembly method (Figure 1) [90] that relies on the long-term supplementation of serum and ascorbic acid in the culture medium. Ascorbic acid, a cofactor of the enzyme prolyl-hydroxylase, facilitates the hydroxylation of proline that is crucial to stabilize the collagen fibrils and their deposition. Secreted ECM components accumulate and assemble within the culture, resulting in a tissue sheet that is sturdy enough to be manipulated with forceps. These cell sheets can be superimposed to generate thicker and planar mesenchymal tissue that can be cultured for several weeks. The self-assembly approach allows the reconstruction of tissues that are histologically and physiologically similar to their *in vivo* counterparts [91]. This method has been successfully used to reconstruct numerous human tissues such as blood vessels [91, 92], bilayered skin [3], bladder [93], adipose tissue [94], heart valve [95], ureter [96], and cornea [97]. It also has been demonstrated that this technique allows to preserve subtle differences in native cells, such as receptor expression patterns, and reproduce pathologies, including psoriasis [98], fibrosis [99], amyotrophic lateral sclerosis [100], inflammation [101], and cancer [102, 103].

### 4.1. Cell Culture and Tissue Production

Cells can be either obtained from nonprofit organizations or isolated from fresh human tissues after obtaining the patient's consent. These tissues are usually collected following elective surgeries (normal skin, scars) or biopsies performed by a specialist. In this case, the biopsy is cleaned with sterile saline containing antibiotics, and then mechanically and/or enzymatically dissociated to digest the ECM and the basal lamina [104–107]. Of particular interest, it is possible to isolate several cell types from a single biopsy to create patient-derived tissue-engineered constructs to study disease-specific microenvironments. Different cell types can be cultured alone or in combination with one another, either isolated from healthy donors or from different patients to decipher the role of a specific cell type in the disease pathological process. To obtain cell and matrix-rich sheets that can be stacked upon one another and easily handled, stromal cells are first seeded on a tissue plate in the presence of a peripheral anchorage device (Figure 1(a)). This support limits the contraction of the tissue and facilitates the manipulation of the resulting cell sheet. Stromal cells are classically cultured in Dulbecco's Modified Eagle's medium containing 50 *μ*g/ml ascorbate, 10% bovine serum, and antibiotics. After 14 to 28 days, several sheets are stacked to form a thicker stromal compartment. Then, and if needed, for skin or bladder reconstruction, for example, epithelial cells can be seeded on the surface of the reconstructed stroma. Afterward, such bilayered tissue constructs are cultured in an epithelial medium containing 50 *μ*g/ml ascorbate, bovine serum, and additives for 4 to 7 days [3]. The construct is next transferred on supportive components to maintain the tissue at the air-liquid interface and further cultured for 10 to 21 days to allow a proper differentiation of the epithelium. Cancer cells or ready-to-seed 3D spheroids, prepared in parallel, can also be added into the tissue-engineered constructs during the reconstruction process (Figure 1(b)) [102, 103].

### 4.2. Addition of Vascular and Lymphatic Microvascular Networks in Self-Assembled Tissues

Initially, the rationale of adding a microvascular network to self-assembled tissues was to accelerate their perfusion by the host vasculature upon grafting. The underlying hypothesis was that grafted tissues that are more rapidly vascularized will not suffer from alterations due to a lack of oxygen and nutrients. To generate tissue-engineered constructs with microvascular networks, the first step is to have access to endothelial cells, which can be isolated from human umbilical vein, neonatal foreskin, and adult skin (reviewed in [108]). Then, endothelial cells must be cocultured with stromal cells or added to the cell sheets during the maturation phase (Figure 1(c)). When seeded on top of stromal cell sheets, they attach randomly to the surface and quickly form clusters within 48 hours. Tubular-like structures with lumens then emerge, forming spontaneous microvascular networks that persist over several weeks *in vitro* [109, 110]. These microvascular networks are functional when grafted. Indeed, prevascularized self-assembled tissue-engineered skin, adipose tissue, blood vessels, and urethra were shown to be perfused within 2 to 4 days following *in vivo* tissue implantation [96, 101, 111, 112]. In contrast, the detection of red blood cells was delayed within the grafts of control tissues without preestablished capillaries (e.g., between 7 and 14 days after grafting). Hence, the presence of a microvascular network accelerates the integration of the grafted tissues.

Of particular interest, prevascularizing reconstructed tissues allows to better understand normal and pathological processes of vascular and lymphatic vessel formation and remodeling, including in cancer. In self-assembled skin substitutes, the epidermis was shown to influence the diameter of dermal capillaries through a process involving the vascular endothelial growth factor [110]. The recruitment, from fibroblasts, of pericyte-like cells expressing *α*-smooth muscle actin or chondroitin sulfate proteoglycan, was also observed [109]. Since pericytes are essential components of the blood microvasculature, the self-assembly method of tissue engineering becomes a powerful approach to study angiogenesis and endothelial cell/pericyte interactions with the stroma *in vitro*. In the field of cancer research, the intima of tissue-engineered blood vessels was shown to react to the presence of the interleukin-1*β* inflammatory cytokine by expressing E-selectin, a binding molecule for circulating cancer cells [109, 113, 114]. Hence, the prevascularization of reconstructed tissues constitutes an interesting tool to investigate normal and tumoral mechanisms.

In many cancers, a common route for metastasis is the invasion of lymph nodes through the lymphatic vessels. It has been shown that lymphatic endothelial cells seeded within tissue-engineered constructs assembled into capillaries exhibiting nearly all of the molecular and ultrastructural features of native human lymphatic microvasculature, including branching in 3D, wide lumen, blind ends, overlapping borders, adherens junctions, anchoring filaments, lack of mural cells, and poorly developed basement membrane [115]. Lymphatics within tissue-engineered constructs also form more tubules in response to lymphangiogenic stimuli [116]. Overall, these models hold promises to decipher the pathophysiology of cancer because the presence of a microvascular network contributes to the process of metastatic progression [117].

## 5. 3D Cancer Modeling Using the Self-Assembly Approach

Cancer modeling is useful for basic and applied research, giving insights into the pathophysiology of tumors and creating novel tools for drug discoveries. As mentioned above, 3D cancer models can be used to study the TME, while 2D culture systems cannot. The understanding of the role of specific fibroblast subtypes, as well as ECM organization, fibre size, orientation, and stiffness, is a crucial aspect in cancer research which has led to the development of novel 3D culture systems using exogenous scaffolds [89]. The heterogeneity and plasticity of stromal cells across the body indeed result in the formation of matrix microenvironments with distinct composition and organization, as illustrated in the papillar and reticular dermis of the skin [8]. These distinctive matrix microenvironments are likely to influence cancer behavior, development, and spreading [118, 119], as shown with the variety of metastatic mechanisms described in prostate [120], colorectal [121], breast [122], bone [123, 124], and skin [125] tissues.

With the development of self-assembly approaches, it has become possible to recreate specific human matrix microenvironments *in vitro*. Self-assembled tissues indeed contain organ-specific stromal cells that provide the proper ECM composition and organization to induce physiological processes, such as cornea [126] and bladder [127] epithelial cell differentiation. When fabricated from pathological cells, self-assembled tissues display abnormal ECM networks resembling native pathological matrices, as shown in reconstructed models of hypertrophic scars [98], scleroderma [128], and psoriasis [98]. In this section, we will show that self-assembled tissues can also mimic the TME of native human skin, bladder, and eye cancers, therefore providing highly valuable study models for cancer research and precision medicine.

### 5.1. Basal Cell Carcinoma

Basal cell carcinoma (BCC) is one of the most prevalent cancers in the world, accounting for 60-80% of all nonmelanoma skin cancers [129]. Most frequent etiologies in patients with BCC include genetic predispositions, chronic ultraviolet (UV) exposure, and nonhealing wounds such as ulcers and severe burns [130]. Usually, BCC manifests in body areas covering the head and neck (80% of cases), the trunk (15% of cases), and the arms and legs. More rarely, it also arises from the armpits, breasts, perianal areas, palms, and soles [131]. Clinically, BCCs are grouped into indolent and aggressive growth forms depending on the patient genetic background. The indolent variants, which are the most common subtypes , include nodular and superficial BCCs, while the aggressive variants comprise micronodular and morpheaform subtypes. Although BCCs are rarely associated with metastases and mortality, they can spread locally and damage the cutaneous tissue, leading to loss of function and disfiguring outcomes [132]. Over the last 50 years, the incidence of BCC has been rising in Europe [133, 134], Asia [135, 136], and America [137, 138], reaching up to 1.5% of the male population in the US. Unsurprisingly, the management of BCC has thus become a significant burden for most healthcare systems. In the US, for example, radiation therapy and surgery treatments can cost over $3,000 per patient [139].

In the mid-19th century, seminal studies, driven by Robert Gorlin, in patients with basal nevus syndrome were the first to suggest a genetic etiology for BCC [140]. Since then, genetic knockout mice havebecome the primary tool to study the disease, based on the fact that BCC patients suffer from a mutation in the Patched 1 (PTCH1) gene, dysregulating the Sonic Hedgehog (SHH) pathway [141, 142]. The first transgenic mice were developed to overexpress the SHH protein [143], or a mutant variant of the downstream protein SMO [144]. However, these mutations were lethal and fostered the development of alternative strategies. The successful overexpression of SHH signaling transcription factors, such as Gli-1 [145] and Gli-2 [146], and the development of UV radiation-sensitive models using PTCH^+/-^ transgenic mice [147] constituted robust *in vivo* models to study BCC. However, considering the distant relationship between rodents and humans, it became more and more questionable whether these models were truly reflecting the effects observed in humans. A few years ago, the SMO antagonist vismodegib, approved by the Food and Drug Administration for the treatment of aggressive BCC, was indeed discontinued because of its cumulative toxicity in patients [148]. This example shows that the use of study models that more closely mimic human physiology is an absolute prerequisite to improve the screening of new BCC therapies.

Over the past few years, discoveries in cancer research have pointed out several factors implicated in the BCC pathophysiology, including mutations in the tumor-suppressing gene p53 [149], activation of the Wnt/*β*-catenin pathway [150], and signaling through the epidermal growth factor receptor [151]. The TME has also been shown to influence BCC growth and progression, through the release of oncogenic proteins such as Gremlin-1 [152] and Galectin-1 [153]. Despite the need for *in vitro* models that recapitulate the human TME and bypass the use of mouse models, there still is a lack of accurate human BCC 3D models, since most of the current research uses animal tissue explants [154] or 2D cell culture.

**Figure 2:**
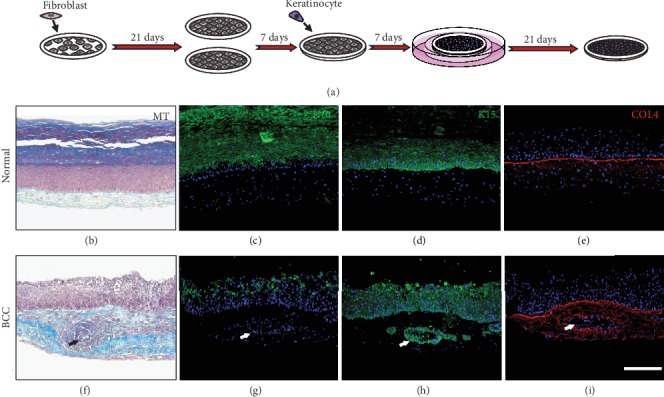
Basal cell carcinoma modeling using the stromal self-assembly method. (a) Experimental procedure: fibroblasts are grown as single-cell sheets, stacked with one another, seeded with keratinocytes, and matured at the air-liquid interface. Characterization of the self-assembled constructs with (b–e) normal cells derived from a healthy individual or (f–i) BCC cells derived from an affected patient. Self-assembled constructs can be analyzed histologically with (b, f) Masson's trichrome (MT) staining or immunofluorescence against epidermal markers, such as (c, g) Keratin 10 (K10), (d, h) Keratin 15 (K15), and (e, i) the basal lamina marker type IV collagen (COL4). (f–i) As shown by the arrows, basaloid nests spontaneously formed in the dermis of BCC constructs. Scale bar = 100 *μ*m.

In this context, the stromal self-assembly method of tissue engineering stands as a promising approach to faithfully recreate the human BCC pathophysiology. Using this method, successful reconstruction of pathological BCC skin constructs has been achieved in 56 days using cells derived from BCC patients (Figure 2(a)). Briefly, fibroblasts were isolated from BCC patients and cultivated with ascorbate until they formed cohesive stromal sheets. These sheets were then stacked to one another, forming a dermal construct, on top of which normal and BCC keratinocytes were seeded and matured at the air-liquid interface. Using this model, BCC skin constructs displayed histological features of BCC cancers, including nests of basaloid cells , surrounded by a fibromyxoid stroma (Figures 2(b)–2(i)). Tumoral keratinocytes also showed abnormal proliferating and nondifferentiating phenotypes compared to the control, as depicted by the divergent expression patterns of K10 and K15 (Figures 2(c), 2(d), 2(g), and 2(h)). The aberrant expression of the basement membrane protein, type IV collagen, around the dermal nests of BCC skin constructs further confirmed the basaloid phenotype of pathological cells (Figures 2(e) and 2(i)).

Although requiring long production times and staff who are well trained in complex cell culture techniques, the stromal self-assembly method of tissue engineering represents a reliable and promising tool to produce human BCC tissues *in vitro*. Compared to other conventional *in vitro* models, the stromal self-assembly method allows the development of a complex 3D TME, essential to recapitulate the histology of human BCC *in vitro*. Additionally, it is neither limited by tissue donor availability nor ethical concerns, as it is often the case with human tissue explant culture models. It can also be used to investigate the role of various targets since cultured cells can undergo any modification before being incorporated into the 3D model.

### 5.2. Cutaneous Neurofibromas

Neurofibromatosis type 1 (NF1) is a common genetic disorder of deregulated cell growth, occurring in about 1 in 3,000 individuals, caused by germline mutations in the neurofibromin-encoding gene *NF1* [155]. People with NF1 are at an increased risk of developing a variety of benign and malignant tumors. There are limited therapies and no cures for NF1. It causes brain tumors, as well as disfiguring skin lesions called cutaneous neurofibroma (cNF) that stigmatize the affected individuals. The morphogenesis of neurofibromas is poorly understood and their formation can highly vary between patients, with significant differences sometimes seen within the same family. The presence of cNFs is one of the main clinical features of patients affected by this disease. Neurofibromin is a tumor suppressor protein involved in the regulation of the RAS/MAPK signaling pathway [156–158]. Typically, cNFs are benign tumors developing from peripheral nerves that form rounded and pedunculated masses within the dermis [159–161]. They are principally composed of Schwann cells, fibroblasts, endothelial cells, and mast cells, all embedded in a dense collagen-rich ECM [162–164]. In addition to cNF, NF1 patients can develop plexiform neurofibromas (pNFs) that can undergo malignant transformation and form a malignant peripheral nerve sheath tumor (MPNST) [159].

The biallelic *NF1* inactivation in Schwann cells is suggested to be the first step of cNF formation, but the exact biological mechanism remains poorly understood [165, 166]. Complete loss of *NF1* function alone cannot explain tumor development, and several experimental studies suggest that other factors such as the *NF1* haploinsufficient cellular type or the stromal microenvironment may also be involved. Clinical presentation of cNFs is highly heterogenous and considerably differs from patient to patient in terms of number, size, occurrence, and localization [167, 168]. In numerous cases, neurofibromas instigate significant disfigurement and discomfort, causing psychosocial problems among NF1 patients. Although significant progresses have been made in the understanding of NF1 biology, patients still face significant morbidity and decreased life span. The pathogenic mechanisms of cNF formation and progression are still largely unknown, and there is no specific treatment for this complication. Traditional 2D cell culture models, as well as NF1-derived animal models, failed to recapitulate this important aspect of the disease manifestations [169–171]. Kraniak et al. have developed 3D models where immortalized NF1-associated cells isolated from pNF formed spontaneously spherical aggregates when seeded in Matrigel [172]. They also tested different drugs and observed resistance to growth inhibition in cells cultured in 3D. Other groups have demonstrated that induced pluripotent stem cell- (iPSC-) derived NF1 Schwann cells formed 3D spheroid-like structures in culture and expressed specific pNF markers [173]. Indeed, these 3D models are particularly interesting and beneficial for the NF1 field, but they are not necessarily suitable for long-term studies. Also, the ECM scaffold used for 3D cell culture does not represent the TME usually found in NF1-associated neurofibromas.

**Figure 3:**
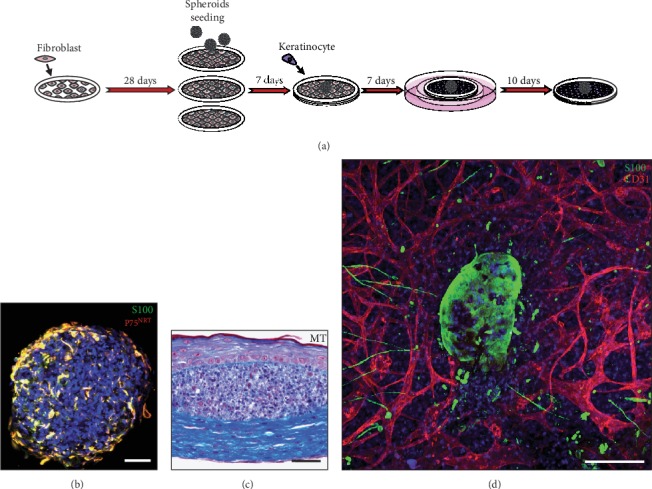
Cutaneous neurofibroma modeling using the stromal self-assembly method. (a) Experimental procedure: dermal fibroblasts isolated from skin biopsies of NF1 patients are cultured for 28 days in the presence of ascorbate to generate cell sheets. Neurofibroma-like spheroids consisting of NF1-associated Schwann cells and fibroblasts are seeded onto the surface of the upper cell sheets. After 3 additional days, 3 cell sheets are stacked and keratinocytes are seeded on the upper side (i.e., on the same side as neurofibroma-like spheroids). One week later, the whole construct is lifted at the air-liquid interface to promote an optimal differentiation of the keratinocytes. If needed, endothelial cells can be added on the 2nd and 3rd cell sheets 7 days before the stacking step, to add microvascular networks to self-assembled tissues. (b) Visualization of NF1-derived Schwann cells in ready-to-seed neurofibroma-like spheroids by immunofluorescence using specific markers, such as S100 and P75NRT. (c) Histological characterization of the NF1 self-assembled construct stained with MT. Neurofibroma-like spheroids formed rounded and well-circumscribed masses that are located at the dermoepidermal junction. (d) Self-assembled constructs can also be imaged in 3D using confocal microscopy to visualize complex structures involving mature microvessel networks (CD31) surrounding the NF1-associated tumor (S100). Scale bar = 50 *μ*m (b–c) and 200 *μ*m (d).

It has been recently shown that patient-derived spheroids, composed of NF1^+/-^ Schwann cells alone (Schwannoma) or cocultured with NF1^+/-^ fibroblast cells, inoculated within tissue-engineered 3D skin substitutes, led to the formation of cNF with histopathological characteristics reminiscent of native tumors [174]. Thus, this innovative patient-derived 3D cNF model could allow to better study neurofibroma morphogenesis and stimulate research on this topic. The well-known hanging drop method was used to generate the abovementioned patient-derived spheroids. Three days before the superposition of 3D fibroblast cell sheets, well-formed spheroids were seeded on the top of the previously generated NF1-derived skin substitutes using the self-assembly method of tissue engineering (Figure 3). The full 3D cNF tissue-engineered model was obtained after 52 days of culture. Interestingly, spheroid composition and cellular concentrations can easily be modulated before the incorporation within the tissue-engineered dermis. Different ratios of each cell type can also be used, and different cell types normally found in neurofibromas, such as endothelial cells and mast cells, can be added within the 3D spheroids to recreate a vascularized TME (Figure 3(d)).

This model can be used to study the cell of origin leading to the formation of cNFs and clinical heterogeneity associated with different gene mutations. Finally, this 3D cNF model could have a considerable impact on the understanding of pathogenic mechanisms underlying NF1 and become anajustable platform to accelerate drug discovery and prevent the development of these tumors.

### 5.3. Skin Melanoma

Melanoma is the deadliest skin cancer. In 2015, its global incidence was estimated to 351,880 cases with an age-standardized rate of five cases per 100,000 persons [175]. According to the National Cancer Institute, there were about 96,480 new melanomas diagnosed in 2019 in the US alone, and 7,230 people are expected to die of this cancer [176, 177]. Cumulative sunlight exposure has been described as the major etiological factor in melanoma [178, 179], and DNA damages that are created by UV radiation are also prognostic for outcomes [180]. Currently, diagnosis at an early stage and surgical ablation of the tumor is the best avenue to maximize patient survival [177].

Skin melanoma originates from the malignant transformation of melanocytes, which are located in the basal lamina of the epidermis and hair follicles. From a histopathological perspective, tumor development in the primary melanotic lesion begins by two successive phases: a horizontal phase, which is characterized by uncontrolled cell growth, followed by a vertical phase, in which EMT eventually occurs, promoting malignancy [181, 182]. Crosstalk between melanoma cells and the TME, including immune cells, further influence metastatic progression, which ultimately results in the invasion of capillaries and dissemination to distant sites.

To study and understand skin melanoma *in vitro*, it is important to recreate the native tissue. In recent years, there have been several attempts to include specific components of the melanoma TME to better study its pathophysiology [183, 187]. Indeed, such models can help elucidate invasive mechanisms of this cancer through its interaction with fibroblasts and keratinocytes [184, 185]. In an interesting report, Morales et al. assessed the sensitivity of melanoma cells to the B-Raf inhibitor vemurafenib in a 3D coculture system consisting of metastatic melanoma cells incorporated in a dermal fibroblast-derived matrix scaffold [186]. In the field of oncopharmacology and drug delivery, basic spheroid models have been used to study the potential of microparticles to deliver drugs [187]. Interestingly, spheroids can also be added to 3D skin models to create moles. Such 3D organotypic skin-bearing melanoma spheroids have been useful to investigate anticancer drugs in an environment that recreates a major part of the complexity of the tumor [186, 188].

**Figure 4:**
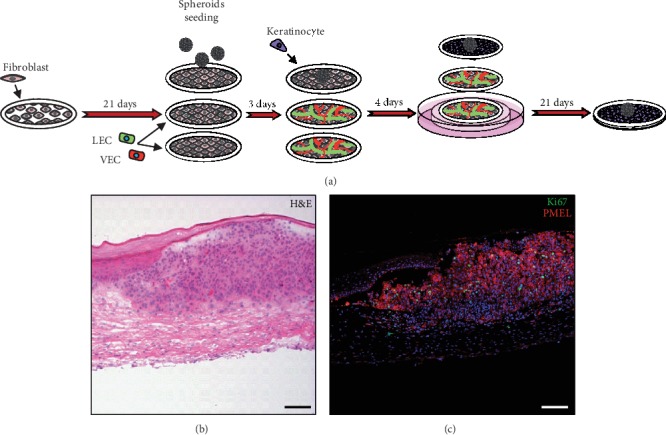
Skin melanoma modeling using the stromal self-assembly method. (a) Experimental procedure: fibroblasts are cultured 21 days in the presence of ascorbate to induce cell sheet formation. Vascular endothelial cells (VEC) and lymphatic endothelial cells (LEC) are then added on the 2nd and 3rd cell sheets, while spheroids and keratinocytes are added on the upper cell sheet. Three cell sheets are stacked after a total of 28 days of culture and are lifted at the air-liquid interface. (b) Haemotoxylin and eosin (H&E) staining of a WM983B melanoma spheroid in the self-assembled construct showing the extent of melanoma cell invasion. This image has been modified from Bourland et al. [102]. (c) Detection by immunofluorescence of WM983B melanoma cells stained with melanocyte protein (PMEL) and with a proliferation marker (Ki67). Scale bar = 100 *μ*m.

The addition of a microvascular network to 3D models is necessary to recapitulate the full extent of the metastatic process. The presence of a microvascular system was shown to be required to trigger the vertical invasion of melanoma cells *in vivo* [117]. Because microvascular networks are not present in the majority of skin melanoma models developed to date, their influence has been investigated using a self-assembly approach. Gibot et al. have reported that an angiogenic effect is induced by the presence of melanoma cell lines seeded randomly in 3D skin models [189]. To further recreate the native heterogenous tumor microenvironment , melanoma cells seeded as tumor spheroids were also added to this model in addition to both vascular and lymphatic capillaries (Figure 4). This recent development allows for the evaluation of potential therapeutics and novel drugs in melanoma progression in a complex environment exempt from exogenous material. Of particular interest, Bourland et al. have investigated the impact of chronic treatment with vemurafenib in the model and observed a dose-dependent response on proliferation and apoptosis [102].

The protocol to investigate melanoma through this approach required 7 weeks. In the first week, cells were thawed and amplified. In the second week, fibroblasts were seeded on a paper anchor and cultured in the presence of ascorbate to produce macroscopic living cell sheets that could be manipulated with forceps. Melanoma spheroids were also produced in the second week, and endothelial cells, as well as keratinocytes, were thawed and amplified. On the 21st day, endothelial cells and melanoma spheroids were seeded onto the fibroblast cell sheets. On the 24th day, keratinocytes were seeded on a few number of fibroblast cell sheets. One week later, two cell sheets without keratinocytes were stacked under a cell sheet seeded with keratinocytes and placed at the air-liquid interface. The complete models were considered mature (stratified epidermis, incorporated tumor, pigmentation, and capillary networks) at day 38, and experimental treatments, such as the exposure to UV radiation, could be investigated. Of particular interest, numerous primary human or metastatic melanoma cell lines are commercially available and can be added as spheroids in the model. The metastatic potential of these cancerous cells can be assessed in tissue-engineered skins with or without the addition of oncogenic and/or prometastatic factors such as UV radiation.

To summarize, 3D skin models of melanoma have the potential to provide relevant information on melanoma progression, invasion, and response to therapeutics. These models may constitute interesting alternatives to animal studies by allowing them to investigate specific questions [183, 187]. Hence, it is reasonable to envision that 3D skin models will be increasingly used as research tools to decipher specific mechanisms related to metastatic progression and the development of therapeutics in melanoma.

### 5.4. Bladder Cancer

Bladder cancer is a recurrent and malignant disease that afflicts around 550,000 patients per year. It is the 10th most common cancer [190]. Bladder cancer affects more men than women with a sex ratio of 3 : 1 [191]. The main cause of bladder cancer is environmental or occupational exposure to carcinogens, especially tobacco [192]. It is characterized by a great level of recurrence and increased aggressiveness upon return.

The bladder consists of four different layers: from the outer to the lumen, (1) the adventice which consists of a connective tissue roughly similar to the adipose tissue, (2) the muscular layer called detrusor which has the function of expelling the urine outside the bladder during the muscle contraction micturition phase, (3) the submucosa layer called *lamina propria* which consists of a connective tissue joining the detrusor and the fourth layer, and (4) the urothelium. The urothelium is the bladder epithelium separated from the rest of the bladder by the basal lamina. It consists of 5 to 7 layers of pseudostratified bladder urothelial cells (BUCs); the basal layer, which contains the stem/progenitor cells and poorly differentiated BUCs; and the intermediate layers, 3 to 5 layers depending of the filling status of the bladder, which consists of elongated cells attached to the basal lamina, the most basal cells being less differentiated than the most apical ones. Finally, the superficial layer is found directly in contact with the urine, and consists of large, flat, frequently binucleated cells. These cells are called umbrella cells and cover several intermediate cells. They express and assemble uroplakins which form plaques of proteins to protect the epithelium from the urine, a toxic and potentially carcinogenic liquid waste. The urothelium is in constant interaction with the underlying *lamina propria* to maintain tissue homeostasis, adequate proliferation rate, and differentiation of BUCs [193–196].

To study and understand bladder cancer, it is important to work with a model exhibiting similar properties to the native tissue. Several assays were made for three-dimensional bladder cancer modeling, especially spheroid and organoid models [197–199]. Recent publications have highlighted the importance of the chosen protocol to produce spheroids [200, 201] and their usefulness compared to two-dimensional cell line cultures [202]. Also, the organoid model of bladder cancer allows demonstrating the involvement of the Wnt/*β*-catenin pathway in cancer progression [203]. Interestingly, long-term three-dimensional models from patient-derived primary cancer cells have been established as powerful models to potentially develop personalized treatment [204–206].

A self-assembly protocol has also been used to model bladder cancer. Originally, the goal of developing a 3D bladder model was to address a need to increase the bioavailability of tissues for bladder augmentation by generating tissue-engineered autologous substitutes. Since numerous reconstruction techniques use biomaterials or exogenous materials to obtain such models, cell differentiation and function may not be physiologically accurate, limiting their grafting potential [107].

The self-assembly technique was used to obtain a completely human 3D bladder model. Stromal cells were used to secrete and form the ECM, and urothelial cells were then seeded directly on the stroma [127]. Fibroblasts from a skin biopsy were generally used to produce these models. Results showed that nonbladder mesenchymal cells altered urothelium differentiation. The urothelial cells presented epithelial markers, such as cytokeratin 14, after they were cultured on the stromal compartment. Therefore, the use of organ-specific stromal cells was tested to improve and normalize the urothelium differentiation and eliminate this problem. The bladder mesenchymal cells, contrarily to dermal fibroblasts, promoted the normal differentiation of urothelial cells, eliminating the presence of epithelial markers. This reconstructed tissue was first produced in a porcine-derived model [127], followed by a human-derived model [103].

**Figure 5:**
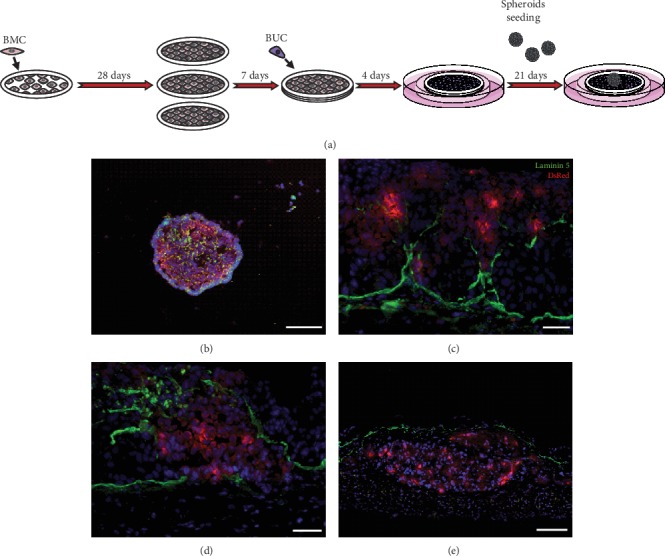
Bladder cancer modeling using the stromal self-assembly method. (a) Experimental procedure: bladder mesenchymal cells (BMC) are cultivated as cell sheets, superimposed, seeded with bladder urothelial cells (BUC), and matured at the air-liquid interface. Once the basal lamina is formed (10 days after the constructs are raised at the air-liquid interface), bladder cancer cell line-derived spheroids are added to the model. (b) Using this model, it is possible to track the fate of the implanted spheroids when DsRed-expressing tumor cells are used. (c–e) It is also possible to visualize over time the basal lamina disruption upon spheroid crossover using Laminin-5-DsRed staining. Scale bar = 100 *μ*m.

To study the development of bladder cancer cells *in vitro*, it is important to reproduce a valid tumor microenvironment. To achieve such a feat, all cells used in the self-assembly technique have to be isolated from a human bladder biopsy. Three sheets of fibroblasts and their ECM were created in separate culture dishes and superimposed to obtain the thickness needed for our 3D model. To promote ECM formation, fibroblasts were stimulated with ascorbate and serum over 21 days. After the stacking of the sheets, urothelial cells were cultured on the surface sheet. After 4 days of culture in medium, the tissue was raised to the air-liquid interface to mimic the conditions in which urothelial cells differentiate for 21 days. Finally, to assess cancer development, spheroids made from cancer cells were deposited on the newly differentiated urothelium (Figure 5(a)). It is important to note that for the cancer model to be relevant, a basement membrane must have appeared between the urothelium and the stroma. The basement membrane plays a great role in cancer progression and epithelial-mesenchymal transition. This detail is often neglected in invasive cancer studies [103].

The obtained model showed many similarities to a native human bladder mucosa. Type I collagen was detected in the stroma, along with CD31, indicating the potential presence of capillary-like networks. Laminin 5 was detected in both the reconstructed model and the native human bladder; it is an important marker showing the presence of a basement membrane. Cytokeratins were also detected in the urothelium of both native and reconstructed tissues [103]. The spheroids made from noninvasive cells did not penetrate beyond the urothelium, just as what would be observed in native tissues, whereas the muscle-invasive bladder cancer cells were able to penetrate the urothelium and completely disorganize the basement membrane. In both cases, the results were similar to what would be seen *in vivo* (Figure 5). It has been proven that it is possible to obtain a self-assembled tissue including the stroma and a differentiated epithelium, separated by a basal lamina. This model is a great tool for tumor progression studies and drug development.

### 5.5. Uveal Melanoma

Uveal melanoma (UM) is the most frequent primary intraocular tumor in adults and is drastically distinct from skin melanoma in terms of etiology, biology, and metastatic organotropism [207, 208]. It arises from the malignant transformation of ocular melanocytes, with a higher occurrence in the choroid coat [207, 209]. The most common age at diagnosis is 50-60 years, and the incidence is highest among people with lighter skin, fair hair, and blue eyes [210]. Familial cases and germline mutations are rare in UM [211, 212]. Key chromosomal abnormalities and driver mutations, as well as the DecisionDx®-UM gene expression profile test allow classifying UM patients at low or high risk of metastasis [213]. Indeed, half of the patients develops metastases mostly in the liver (so-called hepatic tropism). The ocular tumor is generally treated by radiation therapy or eye enucleation [214, 215]. However, metastatic disease is incurable. Contrary to skin melanoma, only a minority of patients benefited from immunotherapy [216, 217].

The self-assembly approach of tissue engineering will be of great interest to define the role of the microenvironment in UM, at both the primary and metastatic sites. A tissue-engineered choroid model was recently generated to address the need for 3D substitutes to study diseases affecting this ocular tissue such as age-related macular degeneration. This model includes choroidal fibroblasts with their endogenous ECM, as well as choroidal melanocytes [218], endothelial cells, and the retinal pigment epithelium, and successfully recaptures the biomechanical properties of the native tissue [219]. This innovative 3D system is superior to previously published *in vitro* models of the choroid, which used synthetic scaffolds or primate choroidal endothelial cells [220, 221]. UM-like spheroids were also seeded in this tissue-engineered choroid model to decipher the pathological contribution of the choroidal microenvironment in UM progression [222] and to eventually provide a valuable tool to improve the treatment of the primary tumor.

The development of new therapeutic strategies to cure the metastatic stage of UM will require a better understanding of mechanisms that allow the stromal liver cells, such as hepatic stellate cells (HSteCs), to create a permissive environment [223]. Once activated into a myofibroblastic phenotype, they can prime the premetastatic niche by remodeling the hepatic ECM as demonstrated in metastatic pancreatic and colorectal cancers [224, 225] and more recently in metastatic UM [226, 227]. Also, their fibrogenesis activity may be unfavorable to the efficacy of several anticancer treatments [228]. It is becoming clear that recreating the liver-specific human ECM, both composition and architecture, as well as hepatic cell-matrix/cell-cell interactions, will be difficult but essential for liver metastasis studies [229]. Human liver organoids were recently developed using iPSCs [230]. However, this approach is technically challenging and too expensive to be used as routine 3D models in both the academic and pharmaceutical industry. It is well recognized that synthetic materials and matrices formed from isolated biological materials cannot achieve the molecular complexity and organization of native tissue or tumor matrices. Since UM patients' histological samples correspond mostly to the macrometastatic disease, an *in vitro* model of metastatic UM will allow characterizing the ECM remodeling at both early and late metastatic stages, in addition to xenograft models [226]. The development of HSteC-derived matrices incorporating metastatic UM spheroids is underway to uncover how the desmoplastic reaction of stellate cells contributes to the notorious therapeutic resistance of UM [218, 231]. The strategy of combining the self-assembled cancer organotypic model and microfluidics to generate cancer-on-a-chip models [232] may eventually greatly contribute to predicting drug-induced responses in metastatic UM.

## 6. Perspectives and Future Directions

In this review, we have discussed current advances in 3D *in vitro* cell culture to study cancer. In particular, we described cancer models engineered to study a variety of tumor types, including those developing in the skin, bladder, and eye tissues. Other important cancer types for which solid 3D tumor models are being developed by various teams include prostate and breast cancers [20, 233–240]. Recent advances for these tumor types include the use of organoids and the incorporation of patient-derived samples (blood derivatives, cells, and/or ECM). The increased use of primary patient-derived normal and cancer cells instead of conventional cell lines for 3D engineering *in vitro* enables the development of more accurate 3D models, retaining the patient and tumor characteristics, and reflecting more appropriately tumor heterogeneity in the population. We highlighted, in this review, the paramount contributions of cell-secreted endogenous ECM components by focusing on the engineering of 3D cell models produced by the self-assembly approach of tissue engineering.

**Figure 6:**
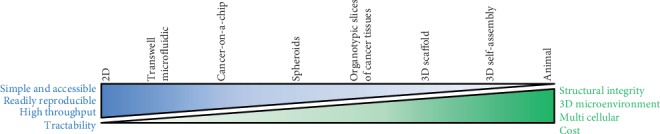
Classification of the most common cancer study models based on their complexity. This diagram highlights the biological (e.g., structural integrity, 3D microenvironment, and multicellular) and technical (e.g., simplicity, accessibility, reproducibility, throughput, tractability, and cost) characteristics of the main study models used in cancer research. Although there is no perfect study model, each of them can be useful and appropriate, depending on the research question to investigate. This classification is simply based on the author's opinion and does not intend to establish new standards.

The 3D cell culture systems are increasingly gaining popularity in tumor and stem cell biology research. To date, numerous 3D cancer models have been specifically developed to take into account the *in vivo* architecture, tumor microenvironments,cell-cell and cell-surrounding matrix interactions, as well as signal transduction in cancer research. These models, ranging from simple 2D monoculture to complex bioengineered tissues, have been developed to better mimic the intrinsic discrepancies in the complexity and functionality of various tissues and tumors (Figure 6). These 3D systems also respectively harbor different intrinsic advantages and limitations and vary significantly in their biological relevance to the *in vivo* situation. More sophisticated 3D systems combining cancer and cells in self-secreted stroma could emphasize the importance of the tumor microenvironment and cell-cell crosstalk in cancer progression and invasiveness. Although each 3D culture technique/model is different in principle, each model shows their advantages and limitations. The selection of one model over another is therefore highly contextual and depends on the studied biological questions.

Among the numerous 3D models, we focused particularly on cancer models developed using the self-assembly method of tissue engineering. This method has been shown to overcome key limitations of currently using 2D flat monolayer cell culture and other simple 3D culture models, including uni-/multicellular spheroids, cancer-on-a-chip, organotypic slices of cancer tissues, and hydrogel- and scaffold-based systems. Indeed, the self-assembly method of tissue engineering allows the production of human/patient-derived 3D organ-specific tumors. As opposed to other 3D models, made with either natural (such as collagen, elastin, and glycosaminoglycans) or synthetic polymers to form various 3D structures, no exogenous scaffolding is needed. The self-assembly method of tissue engineering takes advantage of cell-specific self-synthesized ECM and assembly induced by ascorbate present in the culture media. This scaffold-free method is therefore not liable and not directly interfering with cell behavior, such as migration, proliferation, and aggregation influenced by the various mechanical properties and chemical composition of different bioscaffolding materials [241].

Other advantages of the scaffold-free self-assembly method of tissue engineering include cell-specific secretion of the tumor normoxic or hypoxic microenvironment favorable to cancer stemness through the generation of 3D tissues/tumors with diffusion-limited distribution of oxygen, nutrients, metabolites, and signaling molecules [242]. Enhanced cell survival through strong cell-cell contacts has also been observed using this approach [243]. Quite interestingly, gene-editing techniques such as CRISPR/Cas9 could also potentially be applied to this tissue-engineered approach to generate isogenic tissues to better control eventual experimental procedures.

Some important hurdles are however associated with this technique such as tedious isolation and purification procedures for specific cell types, and long production process and cell culture time requiring frequent changes of culture media by experienced laboratory staff to avoid contamination. Thereby, increased cost over the traditional culture model and large-scale production of the 3D tumor model generated by the self-assembly method could also become a disadvantage. Nevertheless, recent scientific breakthroughs have addressed these limitations using new cell culture protocols aimed at increasing the rate of ECM formation [244, 245]. Furthermore, although many questions and hurdles remain, biobanks of patient-derived 3D cancer models should further refine our understanding of interpatient as well as intrapatient heterogeneity, and hopefully, lead to personalized therapies for cancers.

Increasing the complexity of the microenvironment by creating multicellular niches is bound to further cancer research soon. With our expanding knowledge and control of the parameters required for optimal culture conditions for different cell types, models created *in vitro* will even be more realistic. Engineered 3D models will be useful tools to evaluate the impact of secreted molecules from specific cellular types on the normal and pathological functions. For most types of tumors, the incorporation of immune cells in tissue-engineered models represents one of the next important challenges. Another one consists of the incorporation of the vascular/lymphatic network simulating dynamic blood flow and providing the mechanical signals regulating tumor development and function. An increasing number of studies report the development of such perfusion systems applied to models of various primary and metastatic tumor types [20, 246–249].

Engineered cancer 3D models comprising patient-derived cells have also the potential of becoming valuable drug screening tools and are expected to significantly contribute to the progress of precision and personalized medicine. Indeed, the evidence is pointing out significant differences between treatment responses, partly due to intertumor variation and clonal variation within tumors [250]. New therapeutic approaches are therefore needed to improve current medical practices. Precision and personalized medicines are new concepts, often misused to describe what medicine should resemble in the future [251]. While precision medicine intends to target specific disease variants, personalized medicine is aimed at providing patient-tailored therapies. Examples of precision and personalized medicine include blood transfusion according to blood typing and autologous grafting, respectively. The 3D self-assembled tissues we discussed, which are entirely composed of human cells and matrix, display unique properties as a result of cell donor genetic and epigenetic backgrounds, lifestyle, and medical history. For instance, self-assembled tissues produced from cells of patients with pathological features can be used to evaluate drug efficacy/response in the context of precision medicine.

The 3D cell culture approaches hold a great promise and offer suitable systems for various purposes, ranging from disease modeling to drug target identification drug discovery, disease modeling, and cancer-targeted therapy. Continuous progress in tissue engineering, including the development of various bioreactor systems and 3D bioprinting, will improve the diversity, fidelity, and capacity of 3D culture models in cancer research. Besides the ability to generate geometric constructs containing viable cells, the 3D bioprinting technique may also facilitate high-throughput applications with precise reproducibility [88].
